# Serological identification of Tektin5 as a cancer/testis antigen and its immunogenicity

**DOI:** 10.1186/1471-2407-12-520

**Published:** 2012-11-14

**Authors:** Tadashi Hanafusa, Ali Eldib Ali Mohamed, Shohei Domae, Eiichi Nakayama, Toshiro Ono

**Affiliations:** 1 Department of Radiation Research, Advanced Science Research Center, Okayama University, 2-5-1 Shikata-cho, Kita-ku, Okayama, 700-8558, Japan; 2 Zoology Department, Faculty of Science, Damanhour University, Damanhour, Egypt; 3 Department of Maxillofacial Surgery, Okayama University Graduate School of Medicine, Dentistry and Pharmaceutical Sciences, 2-5-1 Shikata-cho, Kita-ku, Okayama, 700-8558, Japan; 4 Faculty of Health and Welfare, Kawasaki University of Medical Welfare, 288 Matsushima, Kurashiki, Okayama, 701-0193, Japan; 5 Batterjee Medical College (BMC), P.O 6231, Jeddah, 21442, Kingdom of Saudia Arabia

**Keywords:** TEKT5, SEREX, CT antigen

## Abstract

**Background:**

Identification of new cancer antigens is necessary for the efficient diagnosis and immunotherapy. A variety of tumor antigens have been identified by several methodologies. Among those antigens, cancer/testis (CT) antigens have became promising targets.

**Methods:**

The serological identification of antigens by the recombinant expression cloning (SEREX) methodology has been successfully used for the identification of cancer/testis (CT) antigens. We performed the SEREX analysis of colon cancer.

**Results:**

We isolated a total of 60 positive cDNA clones comprising 38 different genes. They included 2 genes with testis-specific expression profiles in the UniGene database, such as *TEKT5* and a CT-like gene, *A kinase anchoring protein 3* (*AKAP3*). Quantitative real-time RT-PCR analysis showed that the expression of *TEKT5* was restricted to the testis in normal adult tissues. In malignant tissues, *TEKT5* was aberrantly expressed in a variety of cancers, including colon cancer. A serological survey of 101 cancer patients with different cancers by ELISA revealed antibodies to TEKT5 in 13 patients, including colon cancer. None of the 16 healthy donor serum samples were reactive in the same test.

**Conclusion:**

We identified candidate new CT antigen of colon cancer, TEKT5. The findings indicate that TEKT5 is immunogenic in humans, and suggest its potential use as diagnostic as well as an immunotherapeutic reagent for cancer patients.

## Background

Colon cancer is one of the major human malignancies. Over the past decade, the overall incidence and resulting deaths have been declining due to early diagnosis [1]. However, a majority of cases still have a poor prognosis even with the advances in chemotherapy and molecular targeted therapy. Recent progress in tumor immunology based on the molecular identification of tumor antigens may allow immunotherapy to become another promising treatment to improve outcomes.

A variety of tumor antigens have been identified by several methodologies. Among antigens, cancer/testis (CT) antigens have became promising targets for diagnosis and immunotherapy for patients with various tumors because of their unique expression pattern [2,3]. The serological identification of antigens by the recombinant expression cloning (SEREX) methodology has been successfully used for the identification of CT antigens. Using cDNA libraries of cancer and normal testis tissues, SSX2 [4], SYCP-1 [5], NY-ESO-1 [6], XAGE-1 [7], and CCDC62-2 [8] have been identified.

In this study, we performed SEREX analysis of colon cancer, and isolated a novel CT antigen, Tektin5 (TEKT5), in addition to a previously defined CT-like antigen, A kinase anchoring protein 3 (AKAP3). Tektins (TEKTs) are composed of a family of filament-forming proteins in the male germ cell-lineage in centrioles and basal bodies and within ciliary and flagellar doublet microtubules [9]. In mammals, five Tektin proteins (TEKT1 – 5) have been identified [10]. We isolated human *TEKT5*, and showed that it has the characteristics of a CT antigen and that it elicits a strong immune response in a subset of patients with cancer, including colon cancer.

## Methods

### Ethics statement

Patient samples were collected in accordance with the Declaration of Helsinki and approved by the Ethics Committee of Okayama University Graduate School of Medicine, Dentistry, and Pharmaceutical Sciences (No. 842).

### Tissues and sera

Tumor specimens and sera were obtained from patients at Okayama University Hospital. Written informed consent was obtained from all patients and healthy donors involved in our study in accordance with the university guidelines.

### Preparation of the cDNA library

mRNA was purified from the colon cancer tissue C164 using a Quick Prep mRNA Purification Kit (Amersham Pharmacia, Piscataway, NJ, USA). mRNA was also purified from normal testicular total RNA. Then, a cDNA expression library was prepared in a γZAP Express vector using a cDNA library kit (Stratagene, La Jolla, CA, USA).

### Immunoscreening of cDNA libraries

**A** cDNA expression library of C164 colon cancer tissues was screened with autologous patient sera. A testicular cDNA expression library was screened with 2 different colon cancer patient sera. The screening procedure was described previously [11]. In brief, serum samples that had been diluted 1:10 were preabsorbed with lysate from *Eschericia coli* Y1090/Y1089 and coupled to Sepharose 4B (BioDynamics Lab Inc., Tokyo, Japan). Recombinant phages (approximately 4,000 pfu) on agar in a plastic dish (140-mm diameter) were amplified for 8 hr and then transferred to 135-mm diameter nitrocellulose membranes (Schleicher & Schuell, Dassel, Germany) for 15 hr at 37°C. The membranes were then blocked with 5% non-fat milk and pre-screened by incubation with peroxidase-conjugated Fc fragment-specific goat anti-human IgG (Jackson ImmunoResearch, West Grove, PA, USA) (1:2,000 dilution) for 1 hr at room temperature. Color was developed using 3, 3'-diaminnobenzidine (Sigma, St. Louis, MO, USA), and IgG-encoding clones were marked so that they could be excluded from subsequent examinations. The membranes were then incubated overnight at room temperature with the preabsorbed serum diluted to 1:200. The membranes were incubated with peroxidase-conjugated Fc fragment-specific goat anti-human IgG (Jackson ImmunoResearch) (1:2,000 dilution) for 1 hr at room temperature, and then the color was developed. Positive clones were collected and subcloned to monoclonality by 2nd and 3rd screenings using 82- and 47-mm diameter membranes, respectively. A randomly chosen negative clone was included in each assay as a negative control.

### Sequence analysis of reacted clones

The clones that reacted positively were subcloned to monoclonality, purified, and excised *in vivo* to pBK-CMV plasmid forms (Stratagene). The plasmid DNA was prepared using the Quantum Prep Plasmid Miniprep Kit (Bio-Rad, Hercules, CA**,** USA). The nucleotide sequences of the cDNA inserts were determined using an ABI 3130x1 Genetic Analyzer (Applied Biosystems, Foster City, CA, USA), and sequence alignment was performed with BLAST software and sequences in the GenBank database.

### Total RNA isolation and cDNA synthesis

Total RNA was isolated from the tumor tissues using an RNeasy Mini Kit (Qiagen, Hilden, Germany). Total RNA from normal testis tissues was obtained commercially (BD Bioscience Clontech, Palo Alto, CA, USA). The RNA (2 μg) was reverse-transcribed into single-strand cDNA using Moloney murine leukemia virus reverse transcriptase (Ready-To-Go You-Prime First-Strand Beads, GE Healthcare, Buckinghamshire, UK) and oligo(dT)_15_ as a primer. The cDNA samples were tested for integrity by the amplification of *G3PDH* in a 30-cycle reaction.

### Quantitative real-time RT-PCR

Two-step real-time RT-PCR was performed using a StepOne Real-Time PCR System (Applied Biosystems). cDNA was synthesized using a High-capacity cDNA Reverse Transcription kit (Applied Biosystems). TaqMan Gene Expression Assays (Applied Biosystems) were used to measure the mRNA levels of *TEKT5* (Assay ID: Hs01025979_m1). mRNA levels were expressed as n-fold differences relative to *G3PDH* (internal standard) and the levels in normal testis (calibrator). PCR was performed using TaqMan PCR Master Mix (Applied Biosystems), and the thermal cycling conditions comprised an initial denaturation at 95°C for 10 min, then 40 cycles at 95°C for 15 sec, and 60°C for 1 min. The parameter Ct was defined as the threshold cycle number at which the fluorescence generated by cleavage of the probe passed above the baseline. The *TEKT5* target message was quantified by measuring the Ct value, and transcripts of *G3PDH* were quantified as an endogenous RNA control using TaqMan human *G3PDH* control regents (Applied Biosystems).

### Gene expression microarrays

Gene expression was examined using Agilent Human 1A oligomicroarrays containing 60-mer DNA probes in a 22K format (Agilent Technologies). Of 19,061 spots, 18,086 are non-controls, and there are 17,086 unique transcript sequences from 15,989 unique human genes. Five hundred ng of total RNA from 3 colon cancer tissues for the test samples and normal colon tissue for the reference samples were used to synthesize labeled cRNA (Low RNA Input Linear Amp Kit, Agilent Technologies) in the presence of cyanine 3-dCTP and cyanine 5-dCTP (Perkin-Elmer Life Sciences, Boston, MA), respectively. Differentially labeled test and reference samples were mixed with Agilent control targets before being hybridized onto the oligomicroarrays for 17 hr at 37°C in a rotating oven. The fluorescence intensities of the targets were detected using a laser confocal scanner (Agilent Technologies), and the resulting images were processed using the Feature Extraction Software, version 8.4 (Agilent Technologies).

### Recombinant TEKT5 protein

TEKT5 was expressed in *E. coli* BL21 using the GST-containing vector pGEX-6P-1 (Amersham Biosciences). cDNA amplification primers Eco-KT-s2 (forward: 5^′^-GGCGAATTCGAGTTTGGGACTACTCAG-3^′^) and Sal-KT-as2 (reverse: 5^′^-ATTGTCGACGGTGTGGCCCACCAGGCGCGG-3^′^) were designed to encompass the entire coding sequence of the gene corresponding to amino acid positions 1–486. The isolated GST fusion protein was purified on a gel filtration column (Hiload 16/60 Superdex 200 pg, GE Healthcare).

### ELISA

Recombinant TEKT5 protein (1 μg/ml) in 0.05 M carbonate buffer (pH 9.6) was absorbed onto 96-well plates (Nunc) at 4°C overnight. GST protein was used as a negative control. Plates were washed with PBS/Tween and blocked with 5% FCS/PBS at room temperature for 1 hr. After washing, serum dilutions (100 μl) in 5% FCS/PBS were added and incubated at room temperature for 2 hr. Plates were washed and incubated with secondary antibody (peroxidase-conjugated Fc fragment-specific goat anti-human IgG, Jackson ImmunoReserach) at a 1/5,000 dilution for 1 hr at room temperature. Plates were washed and incubated with the substrate solution (1,2-phenylenediamine dihydrochloride) for 20 min at room temperature. After the addition of 6 M H_2_SO_4_ (50 μl), the absorbance was determined with a microplate reader (BioRad). A positive reaction was defined as an optical density (OD) value for 1: 400 diluted serum that exceeded the mean OD value of sera from healthy donors by three standard deviations.

## Results

### Identification of colon cancer antigens by SEREX

A cDNA expression library of 1.5 x 10^6^ clones was prepared from C164 colon cancer tissue. Immunoscreening 2.6 x 10^5^ clones with autologous serum yielded a total of 38 positive clones. The nucleotide sequences of the cDNA inserts identified 21 different genes, which were designated as OY-CO-1 to OY-CO-21 (Table 1). OY-CO-3, represented by 10 clones, was identical to *Homo sapiens Dihydrolipoamide dehydrogenase (DLD)*.

**Table 1:** SEREX-defined genes identified by autologous screening of C-164 cDNA library

**Antigen**	**No. of clones**	**Identity/similarities**	**DNA microarray (fold change)**^**1**^		
			**C-164**	**C-1**	**C-29**
OY-CO-1	1	Lectin, galactoside-binding, soluble, 3 binding protein (LGALS3BP)	0.72	0.60	0.46
OY-CO-2	1	Rho GTPase-activating protein 18(ARHGAP18)	4.57	1.44	1.50
OY-CO-3	10	Dihydrolipoamide dehydrogenase (DLD)	1.27	0.55	0.30
OY-CO-4	1	Adenomatous polyposis coli (APC), transcript variant 3	3.61	0.74	3.22
OY-CO-5	1	MRE11 meiotic recombination 11 homolog A (MRE11A), transcript variant 1	2.66	9.02	3.72
OY-CO-6	1	Heat shock protein 90 kDa beta (Grp94), member 1 (HSP90B1)	1.02	2.75	1.25
OY-CO-7	1	Transcription elongation factor B (SII), polypeptide 2 (18 kDa, elongin B) (TCEB2), transcript variant 1	1.52	2.74	1.48
OY-CO-8	1	Wilms tumor 1-associated protein (WTAP), transcript variant 2	1.06	0.35	0.42
OY-CO-9	1	Ribosomal protein L35a (RPL35A)	1.77	1.95	2.31
OY-CO-10	1	Polyglutamine-binding protein 1 (PQBP1), transcript variant 7	1.26	1.27	1.30
OY-CO-11	2	PRP38 pre-mRNA processing factor 38 (yeast) domain containing B (PRPF38B), transcript variant 1	3.03	2.88	2.51
OY-CO-12	2	Coatomer protein complex, subunit alpha (COPA), transcript variant 2	1.26	0.73	0.75
OY-CO-13	1	Keratin 18 (KRT18), transcript variant 1	1.46	1.24	0.66
OY-CO-14	1	No strong homology, retrotransposon MSTP055 mRNA	-	-	-
OY-CO-15	1	Ribosomal protein L13 (RPL13), transcript variant 2	0.92	0.84	1.67
OY-CO-16	1	Partial mRNA for KLEIP (kelch-like ECT2 interacting protein), (KLHLX gene)	-	-	-
OY-CO-17	2	BTB (POZ) domain containing 2 (BTBD2)	0.79	0.84	0.83
OY-CO-18	1	Nuclear receptor interacting protein 1 (NRIP1)	0.81	0.87	0.92
OY-CO-19	1	Ribosomal protein L8 (RPL8), transcript variant 2	1.01	2.63	1.69
OY-CO-20	2	Glutamine-fructose-6-phosphate transaminase 1 (GFPT1)	2.92	1.00	0.59
OY-CO-21	1	WD repeat, sterile alpha motif and U-box domain containing 1 (WDSUB1), transcript variant 3	1.69	1.19	0.76

^1^Gene expressions in 3 colon cancer tissues were compared with normal colon tissues by cDNA microarray analysis.

A cDNA expression library of 1 x 10^6^ clones was prepared from normal testicular total RNA. Immunoscreening of 1.6 x 10^5^ clones with C164 colon cancer patient serum yielded a total of 11 positive clones. The nucleotide sequences of the cDNA inserts identified 10 different genes. Among them, 5 genes: *DLD*, *COPA*, *BTBD2*, *GFPT1,* and *WDSUB1,* were also isolated in the autologous serum screening (Table 2).

**Table 2:** SEREX-defined genes identified by screening of a testicular cDNA library with C-164 serum

**Antigen**	**No. of clones**	**Identity/similarities**	**DNA microarray (fold change)**^**1**^		
			**C-164**	**C-1**	**C-29**
OY-CO-22	1	Ribosomal protein L29 (RPL29)	1.28	0.88	1.58
OY-CO-23	1	Sperm-associated antigen (SPAG7)	1.41	0.51	0.52
OY-CO-24	1	Similar to Laminin receptor 1 (LOC388524)	1.10	1.96	1.93
OY-CO-25	1	Microfibrillar-associated protein (MFAP1)	1.30	1.51	1.35
OY-CO-26	1	Glycine cleavage system protein H (aminomethyl carrier) (GCSH), nuclear gene encoding mitochondrial protein, transcript variant 1	2.48	2.47	0.79
OY-CO-3	2	Dihydrolipoamide dehydrogenase DLD	1.27	0.55	0.30
OY-CO-12	1	Coatomer protein complex, subunit alpha (COPA), transcript variant 2	1.26	0.73	0.75
OY-CO-17	1	BTB (POZ) domain containing 2 (BTBD2)	0.79	0.84	0.83
OY-CO-20	1	Glutamine-fructose-6-phosphate transaminase 1 (GFPT1)	2.92	1.00	0.59
OY-CO-21	1	WD repeat, sterile alpha motif and U-box domain containing 1 (WDSUB1), transcript variant 3	1.69	1.19	0.76

^1^Gene expressions in 3 colon cancer tissues were compared with normal colon tissues by cDNA microarray analysis.

A total of 1.6 x 10^5^ clones from the testicular cDNA library were also immunoscreened with another colon cancer patient serum. As shown in Table 3, 15 positive clones representing 12 genes were isolated, including 2 genes with testis-specific expression profiles in the Unigene database. OY-CO-35, represented by a single clone, was identical to a CT-like gene, *AKAP3*. OY-CO-36, represented by a single clone, was identical to *Tektin 5* (*TEKT5*).

**Table 3:** SEREX-defined genes identified by screening of a testicular cDNA library with C-18 serum

**Antigen**	**No. of clones**	**Identity/similarities**	**DNA microarray (fold change)**^**1**^		
			**C-164**	**C-1**	**C-29**
OY-CO-27	2	Chromosome 5 open reading frame 45 (C5orf45), transcript variant 1	0.72	0.92	1.88
OY-CO-28	2	Palladin, cytoskeletal associated protein (PALLD), transcript variant 2	0.04	0.30	1.89
OY-CO-29	2	Heat shock 70 kDa protein 1A (HSPA1A)	2.79	1.72	0.61
OY-CO-30	1	Peroxisomal D3, D2-enoyl-CoA isomerase (PECI), transcript variant 1	0.11	1.65	0.60
OY-CO-31	1	Phosphatidylinositol-5-phosphate-4-kinase, type II, alpha (PIP4K2A)	1.82	0.14	2.21
OY-CO-32	1	Prostaglandin D2 synthase 21 kDa (brain) (PTGDS)	0.19	0.56	0.19
OY-CO-33	1	No strong homology-sequence from clone RP11-486H9	-	-	-
OY-CO-34	1	Tektin 5 (TEKT5)	3.55	7.96	0.33
OY-CO-35	1	A kinase anchor protein 3 (AKAP3)	1.28	1.50	0.72
OY-CO-36	1	Similar to hypothetical protein MGC:37569	-	-	-
OY-CO-37	1	Polycystic kidney disease 1 (autosomal dominant) (PKD1), transcript variant 1	0.42	0.73	0.76
OY-CO-38	1	Coiled-coil domain containing 19 (CCDC19)	3.23	1.67	0.74

^1^ Gene expressions in 3 colon cancer tissues were compared with normal colon tissues by cDNA microarray analysis.

### Gene expression profiles of SEREX-defined genes

Gene expression analysis was performed on 3 colon cancer specimens including C164 used in SEREX, with a cDNA microarray. Of 38 SEREX-defined genes, ten genes showed higher expression levels in all 3 colon cancer specimens compared with normal colon tissue. *MRE11A* and *PRPF38B* showed more than 2.5-fold expression in all colon cancer specimens. *TEKT5* expression was highly up-regulated in 2 colon cancer specimens (3.55- and 7.96-fold). *AKAP3* expression was also up-regulated in 2 colon cancer specimens.

### *TEKT5* mRNA expression in normal and malignant tissues

**Figure 1:**
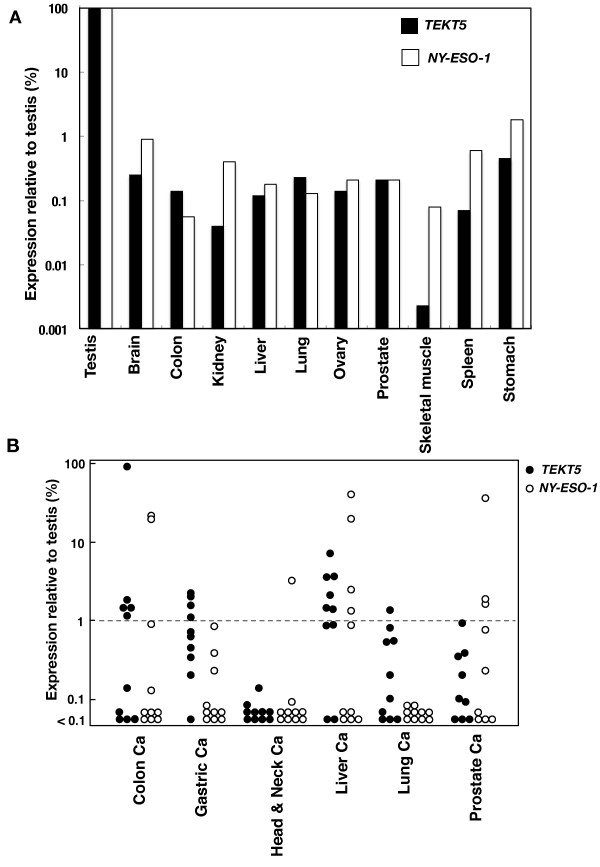
**Quantitative real-time RT-PCR analysis of *TEKT5 *and *NY-ESO-1*.** (**A**) Expression in normal adult tissues. Expression relative to testis is indicated. (**B**) Expression in colon cancer, gastric cancer, head & neck cancer, liver cancer, lung cancer, and prostate cancer.

To investigate *TEKT5* mRNA expression in normal tissues, we performed quantitative real-time RT-PCR analysis using a *TEKT5*-specific TaqMan probe. For comparison, a prototype CT antigen, NY-ESO-1 (TaqMan Gene Expression Assays: Hs00265824_m1) was also analyzed. As shown in Figure 1A, markedly lower levels of the *TEKT5* gene transcript were observed in normal, nongametogenetic tissues compared to normal testis, as in the case of NY-ESO-1. In malignant tissues, *TEKT5* mRNA expression was detected in 5 of 10 colon cancers, 4 of 10 gastric cancers, 6 of 10 liver cancers, 1 of 10 lung cancers, and 1 of 9 prostate cancers at >1% of the testicular expression level. Furthermore, one colon cancer showed an expression level equivalent to the testis (Figure 1B). *NY-ESO-1* mRNA expression in the same set of cancer specimens was also indicated in Figure 1B.

### Immunogenicity of TEKT5 in cancer patients

**Figure 2:**
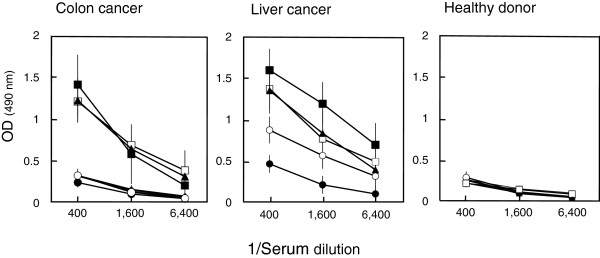
**ELISA reactivity of sera from cancer patients and healthy donors against TEKT5 protein.** Each line represents a titration curve of a serum from single patient.

We then investigated the immunogenicity of TEKT5. Sera from 101 cancer patients and 16 healthy donors were tested for IgG antibody by ELISA using recombinant TEKT5 protein. As shown in Table 4, 4/44 sera from colon cancer patients, 6/15 sera from liver cancer patients, and 3/19 sera from head & neck cancer patients were reactive against TEKT5. No TEKT5 antibody was detected in the sera from 23 lung cancer patients. None of the 16 healthy donor serum samples were reactive in the same test. Figure 2 illustrates titration curves with sera from selected cancer patients and healthy donors.

**Table 4:** Antibody response to the recombinant TEKT5 protein in sera from healthy donors and cancer patients by ELISA

**Sera**	**Positive/total**
Healthy donor	0/16
Colon cancer	4/44
Liver cancer	6/15
Lung cancer	0/23
Head & Neck cancer	3/19

## Discussion

In our study, we performed the serologic search for colon cancer antigens using SEREX methodology. We isolated a total of 60 positive cDNA clones consisting of 38 different genes, designated OY-CO-1 to OY-CO-38. There were 2 genes with testis-specific expression in the UniGene database and literature, such as *AKAP3* and *TEKT5*.

AKAPs are a group of structurally diverse proteins that bind to the regulatory subunit of PKA [12]. They are signaling scaffolds that contribute to various aspects of cAMP signaling [13]. It has also been demonstrated that AKAP-mediated PKA activation inhibited cell growth in the muscle and T lymphocytes. AKAP3 is a testis-specific protein expressed exclusively in round spermatides [14]. Previously, we showed that AKAP3 was a CT-like antigen, and that high *AKAP3* mRNA expression was observed in ovarian cancer and the expression was correlated with the histological grade and clinical stage of the tumor. We showed that *AKAP3* mRNA expression was an independent and favorable prognostic factor in patients with poorly differentiated ovarian cancer [15].

**Figure 3:**
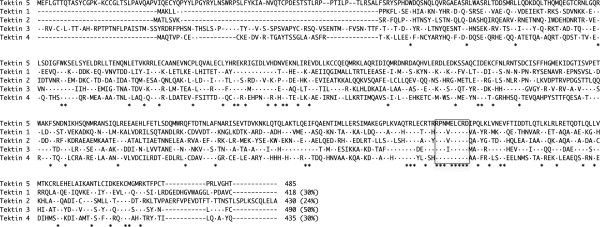
**Sequence alignment of tektin proteins.** Dot indicates the identical amino acid residues in TEKT1 to 4 with TEKT5. An asterisk indicates fully conserved residues. The tektin signature sequence (RPNVELCRD) is boxed. Relative identities of TEKT5 to other TEKTs are 24 to 50% (shown in parenthesis).

TEKTs are composed of a family of filament-forming proteins in the male germ cell-lineage in centrioles and basal bodies and within ciliary and flagellar doublet microtubules [9]. They were originally isolated from sea urchin sperm [16]. Five types of mammalian Tektin have been identified in various animals, including the mouse [17,18], rat [19,20], and human [21,22]. TEKT5 was first identified in the rat [19]. It is present in sperm flagella and plays an important role in flagella formation during spermiogenesis as well as being implicated in sperm motility. The human *TEKT5* gene consists of 7 exons and is located on chromosome 16p13.13. The deduced amino acid sequence of human TEKT5 showed a high degree of identity with the ortholog of the mouse (83%) and rat (83%), respectively. Among 5 human TEKTs, the amino acid sequence is significantly different except in the signature nonapeptide sequence region (Figure 3). Quantitative real-time RT-PCR analysis revealed that the expression of *TEKT5* mRNA was restricted to the testis in normal adult tissues. However, it was detected in several types of cancer, including colon, gastric, liver, lung, and prostate cancer. By cDNA microarray analysis, *TEKT5* showed higher expression levels in 2 of 3 colon cancer tissues compared with normal tissue. It was also up-regulated more than 3-fold in 50% of the lung cancers examined (data not shown). Thus, TEKT5 has a classical feature of a CT antigen. In our survey of 101 cancer patients with several types of cancer, 13 patients produced antibody to TEKT5 protein. No reactivity was observed in sera from healthy donors. In terms of the antibody frequency, TEKT5 appears to have a immunogenic potential as a CT antigen.

Among CT antigens, NY-ESO-1, a SEREX-defined CT antigen, was shown to induce a frequent antibody response in cancer patients [23,24], and strong CD4 and CD8 T-cell responses against NY-ESO-1 were also elicited [25,26]. Taken together, the findings suggest that serologically defined TEKT5 provides a molecular basis for diagnostic and immunotherapeutic targets in cancer patients. Thus, the CD4 and CD8 T-cell responses against TEKT5 will be further investigated.

## Conclusions

We identified candidate new CT antigen of colon cancer, TEKT5. The findings indicate that TEKT5 is immunogenic in humans, and suggest its potential use as diagnostic as well as a immunotherapeutic reagent for cancer patients.

## Competing interests

The authors declare that they have no competing interests.

## Authors’ contributions

TH and AM carried out the molecular genetic studies. SD carried out the immunoassay. EN and TO participated in design and coordination of the research and drafted the manuscript. All authors read and approved the final manuscript.

## Pre-publication history

The pre-publication history for this paper can be accessed here:

http://www.biomedcentral.com/1471-2407/12/520/prepub
